# Minor Review: An Overview of a Synthetic Nanophase Bone Substitute

**DOI:** 10.3390/ma11091556

**Published:** 2018-08-29

**Authors:** Steven J. Eppell, Weidong Tong, James McMasters, Yohannes Soenjaya, Anca M. Barbu, Alvin Ko, Jonathan Z. Baskin

**Affiliations:** 1 Department of Biomedical Engineering, Case Western Reserve University, Cleveland, OH 44106, USA; 2 Department of Otolaryngology-Head & Neck Surgery, Case Western Reserve University, Cleveland, OH 44106, USA; 3 DePuy Synthes Joint Reconstruction, Warsaw, IN 46582, USA; wtong2@its.jnj.com; 4 Department of Biomedical Engineering, University of California Davis, Davis, CA 95616, USA; mcmasters@ucdavis.edu; 5 Sunnybrook Research Institute, Toronto, ON M4N 3M5, Canada; yohannes.soenjaya@gmail.com; 6 Department of Otolaryngology-Head & Neck Surgery, Cedars-Sinai Hospital, Los Angeles, CA 90048, USA; Anca.Barbu@csmns.org; 7 Department of Otolaryngology-Head & Neck Surgery, Henry Ford Hospital, Detroit, MI 48202, USA; alvin.ko@gmail.com; 8 Department of Otolaryngology-Head and Neck Surgery and Facial Plastic and Reconstructive Surgery, Louis Stokes Cleveland VA Medical Center, Cleveland, OH 44106, USA

**Keywords:** biomaterial, bone substitute, collagen, mineral, resorbable, load bearing

## Abstract

Material is reviewed that consists of reconstituted collagen fibril gel mineralized in a manner that produces biomimetically sized nanoapatites intimately associated with the fibrils. This gel is formed into usable shapes with a modulus and strength that allow it to be surgically press fitted into bony defects. The design paradigm for the material is that the nanoapatites will dissolve into soluble Ca^2+^ as the collagen is degraded into RGD-containing peptide fragments due to osteoclastic action. This is intended to signal to the osteoclasts to continue removing the material in a biomimetic fashion similar to bony remodeling. Preliminary experiments in a subcutaneous rat model show that the material is biocompatible with respect to inflammatory and immunogenic responses, and that it supports cellular invasion. Preliminary experiments in a critical-sized mandibular defect in rats show that the material is resorbable and functions well as a bone morphogenetic 2 (BMP-2) carrier. We have produced a range of mechanical and biological responses by varying mechanical and chemical processing of the material.

## 1. Introduction

The ideal solution for reconstructing large bony defects when mechanical strength and stiffness are required is to transfer autogenous vascularized bone flaps using micro-vascular techniques. However, microvascular reconstruction is a large clinical undertaking with the potential for significant risk and donor site morbidity and is inappropriate for the majority of bone defect repair. Autogenous non-vascularized cortical bone grafting (for structural support) is less demanding and risky but is of limited supply and still involves some donor site morbidity. These grafts provide functional needs and are non-immunogenic, but since there is no blood supply and recipient blood vessels are unable to penetrate the dense cortex, most osteocytes do not survive the implantation. This results in necrosis of large parts of the graft [1,2]. Alternatively, freeze dried allograft cortical bone from a non-living donor can provide some function [3]. However, this material requires treatment to reduce the likelihood of an immunological reaction or disease transmission. This treatment reduces biological activity, and like autogenous grafts, cadaveric bone grafts are often not substantially remodeled. Demineralized bone matrix (DBM) is devoid of mechanical properties and does not always elicit a favorable biological reaction [4,5,6]. Other issues with allografts include limited supply; erratic performance, perhaps due to varying processing techniques and donor age [5,7,8]; and the risk, albeit small, of disease transmission.

Several synthetic bone substitutes are available but have various deficiencies. Titanium, an effective material for some craniofacial defects, is well tolerated by the body and can support a load. However, it is best used in combination with other material such as autogenous grafts or flaps. When used alone to bridge a defect, particularly in a load-bearing site, the risks of infection, extrusion, fracture, and other complications (e.g., stress shielding) exist as long as the material remains in vivo. Ceramics and bioactive glasses can be effective bony in-fill materials but suffer from several limitations. For instance, it is a matter of debate as to whether sintered hydroxyapatites (HA) are significantly resorbed at all [9,10,11,12,13], and tricalcium phosphate resorbs too quickly to allow for coupled bone formation [11].

The difficulty in reproducing the physiologic and mechanical behavior of bone is related to the complex interactions between bone matrix components and the surrounding cellular and soluble molecule milieu. Mechanically, bone’s structural interdependence on inorganic and organic phases at the molecular scale allows a range of stiffness, strength, and toughness that is uncommon for materials with such high mineral content [14,15]. Mechanical models of bone demonstrate the essential role of nanoscale mineralites with a specific orientation relative to the collagen matrix to achieve the mechanical properties of bone [16,17]. The 3D geometry of bone substitute materials is important [18]. This notion is presented to contrast our approach with others that focus more on duplicating the chemical constituents of bone (mainly apatite and collagen) without much focus on the nanoscale geometry of the constructed composites.

Multiple strategies exist in the literature seeking to produce a biomimetic bone substitute where nano-scale apatites are intimately interdigitated within and among collagen fibrils. Gower et al. have been working toward the use of a process they coined “polymer induced liquid polymerization” or PILP [19] that has been applied to mineralization of collagen fibrils [20] for over a decade now [21]. Another group in Japan has been working almost as long to simultaneously mineralize collagen fibrils while they are being reconstituted [22]. This material is then isostatically pressed into usable shapes [23,24,25,26]. Our group has also been working on mineralizing collagen fibrils [26], although our approach involves allowing the fibrillogenesis to complete before mineralization begins [27]. We then use a constant composition system [28] in an attempt to drive mineralization beyond the levels easily obtainable by simply raising the pH of calcium phosphate solution above its saturation limit. This technique involves using two solutions: one with CaCl_2_ and NaCl, the other with K_2_HPO_4_ and NaHCO_3_. The solutions are placed in separate burettes, which are dispensed via a computer controlled automated titrator. The system utilizes pH as the control parameter. As apatitic mineralites form, basic carbonate groups become incorporated in the mineral precipitating out of solution, resulting in a drop in pH. By detecting small drops (~0.003 units) in pH and then simultaneously releasing the two solutions in a controlled ratio, the ionic concentration is kept constant, thus maintaining a constant molar composition of the reactants.

Design of a bone substitute requires balancing mechanical and biological (osteointegrative) properties. To create a material using biological molecules that has sufficient mechanical strength to function in load bearing applications, the material must be densified. This is done at the expense of porosity, thus impeding osteointegration. A central assumption of our design is that replicating key structural features of bone on a nanoscale relaxes the microscale pore size requirements necessary for osteointegration compared with currently used bone substitute materials. Our preliminary data suggests this is true. A putative mechanism for this result involves activating a cellular remodeling process, as opposed to a chronic inflammatory reaction, thus initiating a primary bone healing mechanism within the implant. It is thus hypothesized that by constructing this bone substitute material as a biomimetic composite, the material will have the potential to function clinically as an off-the-shelf bone implant. Importantly, the implant is designed with the intent of being used to reconstruct some types of defects in aspects of the skeleton that have load-bearing function. Biologically, there is evidence in the presented work that it is replaced with normal lamellar bone in rats, providing justification for further preclinical trials in larger animals.

What follows is a review of work we have previously published on the development and behavior of this material, along with some new data on its in vitro characterization. This is the first time we have presented the full nanoscale biomimetic design rationale for the material. In addition, it is the first time we have presented the physico-chemical, in vitro, and in vivo results in one place.

## 2. Material Designed around Interaction with Osteoclasts

**Figure 1:**
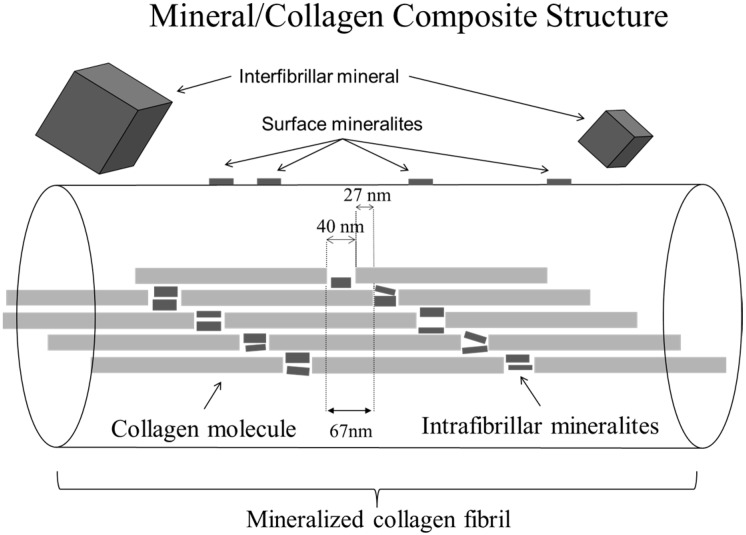
Schematic of fibril made of collagen molecules showing locations for mineral component. Not shown are minerals in the long thing pore zones between collagen molecules, since these only seem to become populated after many months to years in the body [37]. The nanophase bone substitute described in the current manuscript seeks to duplicate mineralization in the 40 nm long gap zones within the fibril. While the larger interfibrillar mineral deposits may also exist, these are not expected to contribute significantly to the behavior of osteoclasts upon material resorption.

Our goal is to produce a bone substitute material mechanically robust enough to provide load-sharing, if not load-bearing, structural support in the defect volume while still being resorbable. Rather than resorption via simple hydrolysis (as occurs in polylactic acid/polyglycolic acid, PLA/PGA, and tricalcium phaosphate, TCP, materials), we aim to have the material removed via osteoclast acitivity at a rate driven by the normal bone-healing process, much like how normal bone-turnover occurs during remodeling. Past work shows that osteoclasts possess membrane-bound receptors optimized for sensing soluble calcium and RGD containing denatured collagen fragments [29,30,31]. These receptors are hypothesized to act in shutting down the resorption activity of the osteoclast [32,33,34]. If this happens too quickly, the biomaterial will not be resorbed. If it happens too slowly, the biomaterial will be removed too quickly for subsequent osteoblastic activity to fill in the resorption vacuole. Thus, biomimetic material must decompose during osteoclastic resorption in a manner that increases the local calcium and denatured collagen concentrations at a rate similar to bone. Local lowering of extracellular pH by osteoclasts provides a physical mechanism for dissolving apatite. However, it has been shown that the nanoapatites in bone will dissolve and reprecipitate even near neutral pH once they are removed from their collagen matrix [30,33,35,36]. Thus, it is possible that natural rises in [Ca^2+^] during remodeling come from a combination of lowered pH and destruction of the protective collagen fibril matrix surrounding the nanoapatites. Our central paradigm for biomaterial synthesis is that the rate of release of collagen fragments and Ca^2+^ ions is controlled by the composite structure of the mineralized collagen fibril in bone. The phases of interest in this composite are the bone apatite and the collagen. The apatite may be found as nanomineralites within the collagen fibril, on the fibril surface, or as larger mineral pieces not directly associated with a fibril (Figure 1). Mimicking the portion of this structure involving nanoapatites directly associated with collagen fibrils thus becomes essential in producing a biomaterial that will be remodeled like the native bone surrounding it. If this mineralized fibril can be duplicated, then osteoclastic action is expected to produce soluble calcium and RGD-containing collagen fragments at a biomimetic rate. Based on the argument above, these components, presented to the osteoclastic ruffled border at the proper ratio, will drive the osteoclast to continue resorbing the synthetic bone.

Obtaining 10 nm sized mineralites and interspersing them with <100 nm spacing within a collagen matrix is difficult. We have accomplished this task by constructing a template for mineralization utilizing the natural self-assembling properties of collagen molecules [26]. The resulting structure has the same molecular arrangement as bone on a <1 μm length scale and possesses 1–10 nm sized void spaces of two shapes: short and thick, long and thin. We fill these voids with solid mineral by thermodynamically driving soluble Ca^2+^, PO_4_^−^, and CO_3_^−^ ions to nucleate inside the collagen fibrils and grow into mineralites.

## 3. Material Fabrication

Fabrication of our material has been described previously [26,27,38,39], and so the presentation here is limited to a brief overview. We use the warm start method [40] with commercially available calf skin collagen. Briefly, a solution of lightly pepsin digested and acid solubilized collagen monomer (3 mg/mL PureCol, Advanced BioMatrix, San Diego, CA, USA) at 4 °C was diluted to 0.2 mg/mL in a TES buffer (Fisher Scientific, Hampton, NH, USA) containing 140 mMol NaCl and 1 mMol potassium phosphate at 37 °C and pH 7.4. After 24 h of incubation at 37 °C, fibril gels were transferred to a metastable calcium phosphate solution similar to a previously published simulated bone fluid substitute [41] without the magnesium (Na 141 mM, K 5 mM, Ca 2.5 mM, HCO_3_ 4.2 mM, Cl 144.8 mM, and HPO_4_ 1 mM) at pH 8.2 to initiate apatite precipitation. Finally, the mineralized gels were rinsed in ethanol, stocked into a cylindrical bore uniaxial press and compressed under either 35 kPa or 350 kPa for 24 h. These samples are referred to as uniaxially pressed (UP) below.

## 4. In Vitro Material Characterization

### 4.1. Sub-µm Structure and Chemistry of Our Material

**Figure 2:**
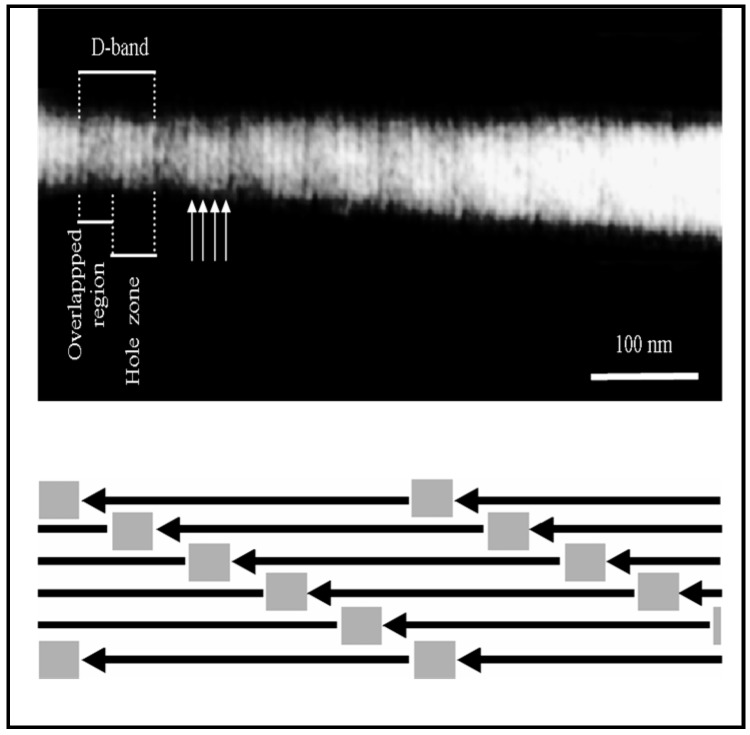
One of our synthetically reconstituted fibrils prepared using uranyl acetate negative staining image using transmission electron microscopy. The high frequency pattern indicated by the arrows is a typical sub-banding pattern seen with this preparation. When four of these bands are averaged, a repeat of 67 ± 4 nm is found. The presence of the banding indicates that our fabrication process results in fibrils with ~40 nm gaps between molecules, which are available for subsequent mineralization.

**Figure 3:**
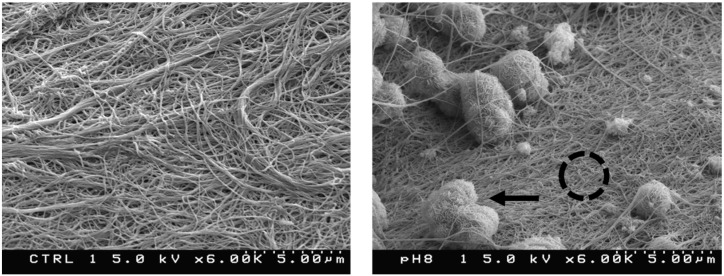
Scanning electron micrographs of our collagen gels before (**left**) and after (**right**) mineralization. Average fibril diameters show a mean of 75 ± 21 nm (*N* = 70). After mineralization, micron size aggregates are visibly trapped in the gel (indicated by arrow in right panel). Energy dispersive X-ray analysis (EDX) shows Ca and P in a ratio of ~1.8 present in these aggregates. On regions of the surface with no apparent aggregates (ex. inside the dashed circle near the middle of the right panel), EDX shows Ca/P ≅ 1.4.

**Figure 4:**
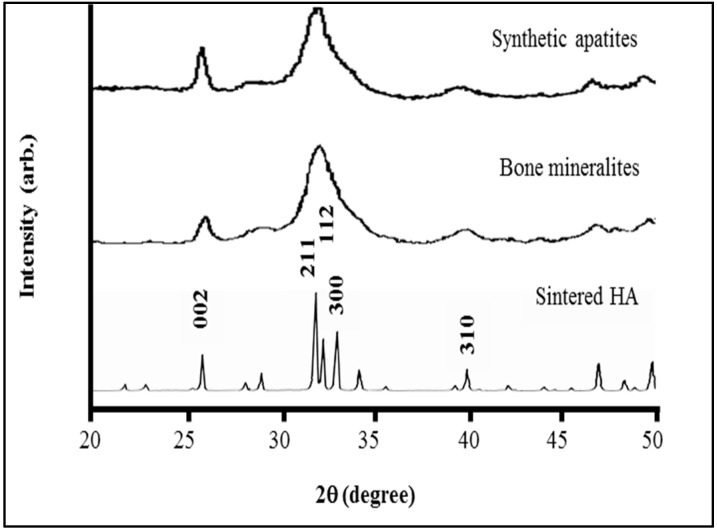
X-ray diffractogram showing synthetic mineral extracted from our material is crystallographically similar to mineral from bovine bone.

Our design requires that the monomers assemble into a D-staggered array [42] containing 40 nm gaps between the amino and carboxy termini of adjacent collagen monomers. Figure 2 shows our synthesis procedure results in such a structure. At lower magnification, scanning electron microscopy shows these banded fibrils have diameters of 75 nm ± 5 (*N* = 70) (unless otherwise stated, all ± values represent the 95% confidence intervals for stated parameters). As seen in Figure 3, the fibrils are entangled with each other. When hydrated, they constitute a loose hydrogel with a density that appears homogeneous to the unaided eye. After mineralizing in a supersaturated solution containing carbonate and phosphate, SEM shows aggregated objects around a micron in size. Energy dispersive X-ray analysis is consistent with these aggregates being apatites; they have Ca/P ratios in the normal bone range of 1.4 to 1.8. Mineral deposits within the gap zones of the D-staggered array would not be visible using SEM. To examine these very small mineralites, we performed a hydrazine extraction and examined the resulting powder using X-ray diffraction to qualitatively assess the crystal perfection as well crystallite size. While intensity characteristics of apatites at angles around 27°, 30.5°, −33.5°, and 40° were observed, no triplet peaks (211, 112, 300) were observed, suggesting that the apatites were very small (Figure 4). While generally the X-ray diffractogram looked very similar to young bovine bone, one can easily differentiate that both the synthetic and natural mineralites differ from sintered HA. The Sintered HA typically has highly crystalline hydroxyapatite and larger crystal size, in which triplet peaks are observed between 30.5 and 33.5 degrees (2 theta). The synthetic apatites have a slightly sharper/taller 002 peak than the natural mineralites. While the mineral/grain sizes are comparable (based on broadening of the 211, 112, and 300 peaks), it is possible that natural bone mineralites have more crystal imperfections such as substitution ions (Na for Ca and Cl for OH), which produce a weaker 002 peak. Collection of multiple XRD patterns would allow us to further characterize the diffraction behavior by providing an average and standard deviation of the 002 peak. In addition, chemical analysis and solubility product (Ksp) may help establish these subtle differences in future research.

**Figure 5:**
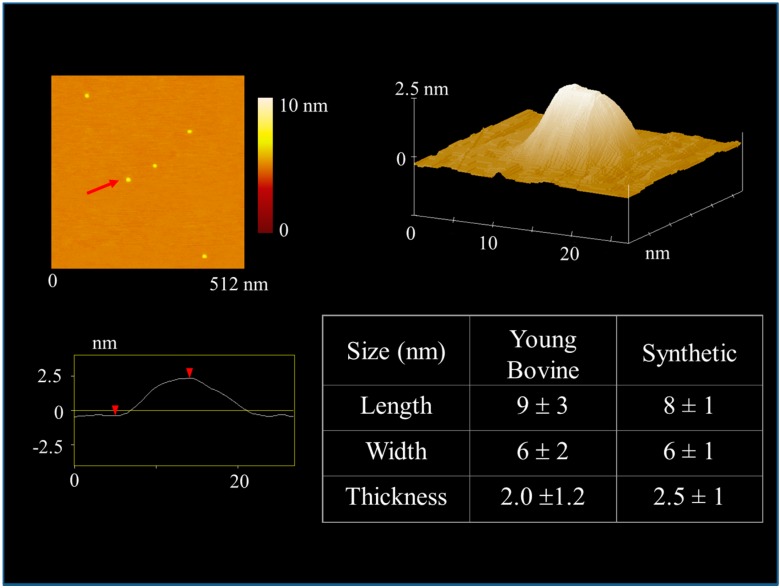
Atomic force microscopy was used to measure the size and shape of natural and synthetic mineralites. Panel at upper left shows top down view of hydrazine-extracted synthetic mineralites deposited on mica. A line scan through the mineralite indicated with the arrow is shown at lower left. The means and standard deviations of mineralites from 1–3 month old cows (*n* = 238) and synthetic mineralites (*n* = 202) are shown at lower right. The upper right image shows a 3D view of the mineralite indicated with the arrow in the upper left panel. This 3D image was typical of both natural and synthetic mineralites.

To visualize individual mineralites within the powder, we used atomic force microscopy (Figure 5). While there were a few (<1%) objects >100 nm in size, the great majority of mineralites had mean dimensions of 8 × 6 × 2.5 nm. These dimensions were similar to those previously measured for young bovine bone [37] with the natural and synthetic dimensions overlapping within one standard deviation (*n* > 200). While mineralites of this size and shape would likely fit into the gap zones inside an assembled collagen fibril, it is not clear at this point if the mineral exists predominately on the surface of the fibrils or if it is also in the interior of the fibril. Tomographic TEM would be able to resolve this issue [43,44].

### 4.2. BMP-2 Release

We looked at the release of BMP-2 from our material incorporating the growth factor in two different ways: [38] (1) We put the BMP-2 into solution during fibrillogenesis (group A); (2) After fibrillogenesis was complete, we immersed the material in a solution containing BMP-2 (group B). Both treatments exposed the material to the same concentration of BMP-2 for the same period of time. Three samples of each treatment were tested. Over the next three days, the material was immersed in DI water, which was exchanged for fresh water 15 times. Just prior to each exchange, we extracted two aliquots and tested for BMP-2 using an ELISA assay. After the first four washes (~12 h), there was no further significant release of BMP-2 from group B materials. Group A materials showed significant BMP-2 release after 10 washes. Thus, incorporating BMP-2 during fibrillogenesis appears to cause a slower release compared to incorporation after collagen fibrils are formed.

For both treatments, the last three measurements showed that the BMP-2 concentration had dropped below the detectable limit of the assay. Thus, any BMP-2 left in the material was not able to be released by simple diffusion. We then treated the material with collagenase, causing complete material degradation. After collagenase treatment, the BMP-2 signal increased for both treatment groups. However, it spiked back up only to the level of the first fluid exchange for the group B samples. Group A samples spiked >5× higher than the original concentration. This is very encouraging and suggests that incorporating BMP-2 during fibrillogenesis may allow for substantial reductions in the amount of growth factor needed in vivo. In addition, incorporation during fibrillogenesis may provide for a more sustained release kinetic, resulting in a more favorable physiological response needed to ensure the implant material is completely resorbed and replaced by bone as clinically required.

### 4.3. Cellular Response

A co-culture of mesenchymal support cells (ST2 from the Riken cell bank) and hematopoietic progenitor cells from young mouse spleens (all animal studies were performed in accordance with the Institutional Animal Care and Use Committee (IACUC), Case Western Reserve University) was used to obtain an osteoblast/osteoclast population on our material [45,46]. Cells were also plated on a few samples of elephant ivory as a positive control for osteoclast formation and activation. We routinely saw resorption features on the ivory similar to those previously reported [47]. Cultures were performed three separate times on three different sets of substrates. The duration of the culture was 10 days.

**Figure 6:**
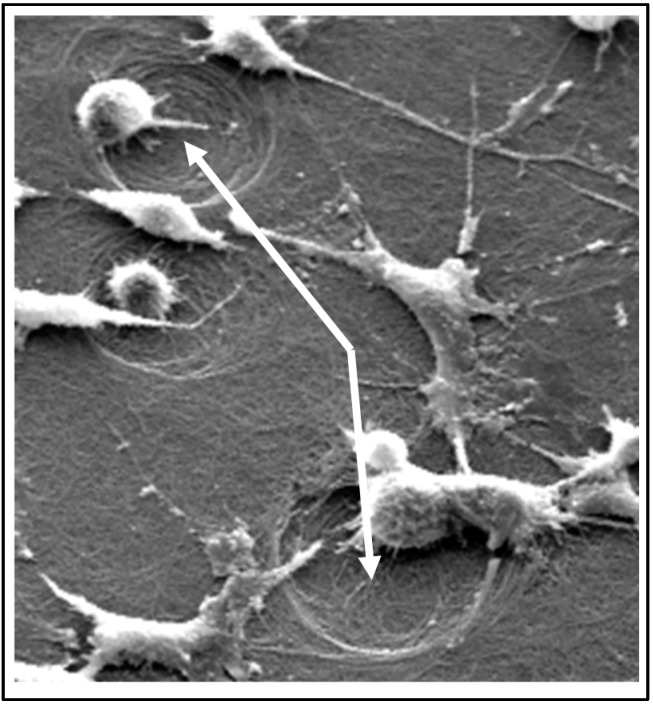
Surface of mineralized collagen gel after 10 days of ST2/osteoclast co-culture. Resorption pits are indicated with arrows. These features were seen only after cell culture.

In every case, the ST2 cells adhered and grew to confluence on our material. In addition, the mouse spleen cells stained positive for tartrate-resistant acid phosphatase (TRAP) and were present as multinucleated cells. We compared the behavior of these osteoclasts on our material with their behavior on unmineralized collagen gels and sintered hydroxyapatite. We looked for resorption pits like those shown in Figure 6. More than 300 randomly chosen fields of view were collected with SEM on each sample type. Pits were counted by eye using a blinded set of files (i.e., the counter did not know from which sample the image under study was collected). Three criteria were used to identify a region of interest as a resorption feature. First, the feature in question was to be 3/4 or more surrounded by a border (a step change in contrast). Second, the bordered area was to be at least 10 μm in its longest dimension. Third, the surface roughness (as measured by the standard deviation of the histogram of image intensity levels) within that border was to be 40% greater than the local surface outside the border. The same size region was used to evaluate the histogram inside the border and on the background adjacent to the border. The 40% threshold was chosen, because the background never varied more than 40% from spot to spot. We expect that the increase in roughness within a resorption feature was the result of collagen fibril disruption during the resorption process. The number of resorption pits per osteoclast on our material was >2× higher than on either the unmineralized collagen or the sintered hydroxyapatite. This finding encouraged us to move on to small animal studies.

The material tested in vitro was an unpressed gel. We were able to substantially densify the gel by mechanical pressing alone. The mechanical properties of this pressed material are sufficient for press fitting samples into custom made defects in bone. However, the samples are not strong enough to hold a screw and they do show substantial loss in stiffness when immersed in water for 30 min. We found that crosslinking the pressed samples allowed them to hold a screw and reduced the loss of mechanical properties upon hydration. Thus, when we moved on to in vivo testing, we used crosslinking as one of our test variables.

## 5. In Vivo Testing

Using a subcutaneous rat model, a study was done with four objectives [39]: first, to establish biocompatibility with respect to immune and inflammatory responses; second, to study the host-material interface as a function of pore size and crosslinking; third, to assess cellular penetration as a function of pore size and crosslinking; and fourth, to compare glycation (d-ribose) and dialdehyde (glyoxal) mediated crosslinking. Glycation mediated crosslinking is found in biological systems and has been studied specifically with respect to collagen [48], whereas dialdehydes are toxic in the unbound state. Seven variations of the material, created by varying the degrees of densification and crosslinking, were tested. A two week time course was used to evaluate the tissue reaction after the acute phase of inflammation had been largely resolved (based on a pilot study) but before any significant material degradation had occurred. Parameters studied included gross morphological features, as well as semi-quantitative histological measures. Histological evaluation was performed by two blinded graders. Foreign body giant cells (FBGC) were counted per high powered field of view in four different fields and averaged. The host-biomaterial interface was assessed by the degree of fibrous encapsulation, which was graded on a scale of 0–4 (measuring the degree of encapsulation and integration of host tissues with the substance of the material) and cellular penetration that was quantified by assessing how far into the material cells had penetrated. All materials were tested in each of 12 rats that necessitated paired statistics. The Wilcoxon signed rank test was used to detect a difference between groups.

**Figure 7:**
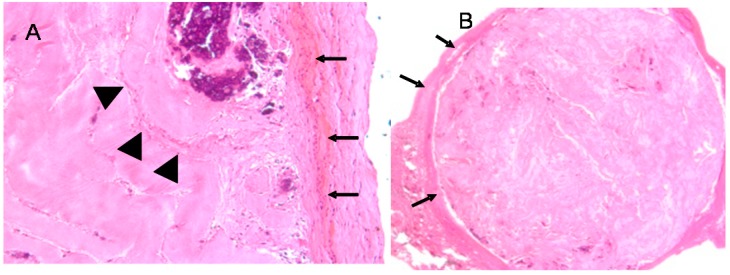
Histological evaluation using an H&E stain of two different preparations of the material. (**A**) non-crosslinked sample. Note the tight adherences of the host tissue with the implant (arrows). While there is evidence of the rudiments a fibrous capsule, the border between host tissue and implant is poorly demarcated. There is a column of fibroblasts and possibly endothelial cells infiltrating into the substance of the implant (arrow heads) 10×. (**B**) a crosslinked sample. Note that there is a well-formed fibrous capsule commonly seen with crosslinked samples (arrows). The capsule did not tightly adhere to the sample, and there was minimal cellular infiltrate 4×.

In each rat, all materials were well tolerated. There was no evidence of gross inflammation, infection, or extrusion. Histologically, the acute inflammatory phase had mostly resolved in all samples, as indicated by the relatively small numbers of polymorphonuclear cells, and there was little evidence of chronic inflammation. FBGC were more ubiquitous in non-crosslinked samples than crosslinked samples. This appears to be a function of material degradation, since a collagen sponge (employed as a control) elicited the highest numbers of FBGC, though without any gross inflammation. In non-crosslinked samples, the material/tissue interface was ill-defined with fibroblasts and likely endothelial cells infiltrating into the bulk of the material (Figure 7) as opposed to crosslinked samples, which caused the formation of a dense and poorly adherent fibrous capsule. Pore size in uncrosslinked samples did not significantly impact the host-material interface using any of the tested metrics. The crosslinked samples had significantly less cellular penetration and soft tissue integration than uncrosslinked samples. Crosslinking time (presumably related to crosslinking density) correlated with degree of fibrous encapsulation and cellular penetration. These results indicated that smaller pore volumes did not negatively impact the biological interface. It further showed that the heavy crosslinking, though effective at stabilizing material constructs, tended to negatively impact the biological interface. Since there was no significant difference between d-ribose and glyoxal with respect to construct stability, d-ribose is exclusively used to crosslink our samples.

## 6. Rational for the Rodent Mandibular Model

The importance of small animal studies in the investigation of bone substitute materials is well documented [49,50]. Our study involved manipulation of craniofacial tissue on a minimal level. Primitive responses of bone to basic functional craniofacial skeletal manipulations are shared by all mammals. Therefore, the rat, as a generic animal model, is appropriate for our level of hypothesis testing [51,52]. Mature males were used, because their size facilitated surgical manipulation of the mandible.

**Figure 8:**
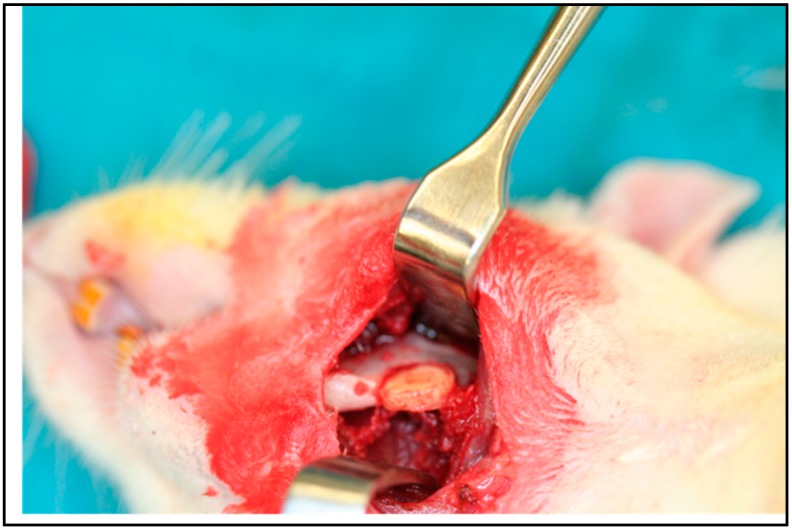
The nanocomposite is implanted in a CSD in the left mandibular body. The material is solidly fixed and well stabilized with solid cortical bone superiorly, anteriorly, and posteriorly. The implant is not easily dislodged on manual palpation.

**Figure 9:**
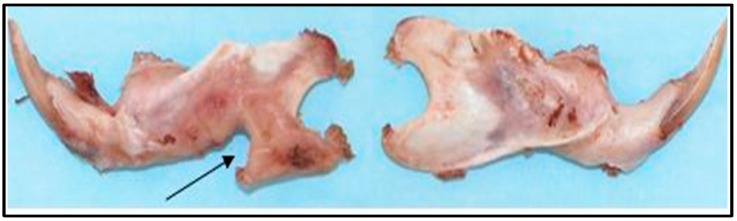
Explanted mandible (dissected free of soft tissue) of a control animal (no implantation) at the conclusion of an 8 week study. ON the left is a left hemimandible following the creation of an unimplanted critical size defect (arrow). On the right is the unoperated contralateral side.

The mandibular critical size defect (CSD) lends itself to testing load-bearing bone substitutes. A critical size defect (>5 mm × 5 mm) is made through robust bone in the body of the mandible. Previous reports suggest the bone near the angle of the mandible is thin, making implant placement and immobilization difficult [49]. We find placing the defect more anterior (rostral) in the mandibular ramus so the anterior extent of the defect lies just postero-inferior to the incisor root allows the defect to be readily extended to 6 mm × 6 mm and beyond while leaving intact surrounding bone that is strong enough to hold an implant firmly in place (Figure 8). This is done without adversely affecting the root of the incisor. When placed in this fashion, rats tolerate the surgery well with no ill-effect to their dentition or eating habits by postoperative day 3 (*n* > 30). This defect does not significantly remodel when left unfilled, which has been cited as a concern in other reports (Figure 9) [49]. The implant is fixed in the defect by creating a precise size match between the implant and defect. Effective fixation of the implant has been demonstrated multiple times by μCT and explantation.

## 7. Nanocomposite Material as a Bone Substitute and Drug Delivery Vehicle

Based on our experience, as well as a review of the literature, a scaffold material alone, even with good osteoconductive properties, will not likely function as an optimal bone substitute without the addition of bioactive factors. This conclusion was confirmed in our material after several renditions were studied using the rat mandible model without the addition of any bioactive factors. Each material was tested in pilot studies with 3 animals per group.

Non-crosslinked samples were resorbed in 8 weeks and appeared to contribute to some skeletal remodeling. However, these samples did not show evidence of osteoinductivity, and the defects were not completely bridged even after all material was fully resorbed. Heavily crosslinked samples were not significantly resorbed. Histologically, a fibrous capsule formed between the material and bone, indicating a fibrous non-union.

Based on these findings, materials were doped with BMP-2. Previous studies showed favorable pharmacokinetics when BMP-2 was used in collagen-based delivery systems [53]. It is not ideal to release high levels of BMP-2 in one initial bolus, as this can result in abnormal bone formation such as a peripheral “bone shell” [54,55,56]. Collagen carriers are associated with sustained release of BMP-2 in contrast to mineral based carriers, which show a high initial release that tapers quickly [57]. Furthermore, ceramics and dehydrated bone can alter the chemical structure of released growth factors [58]. In contrast, BMP-2 binds to collagen sponges without compromising activity, and protein incorporation into the collagen carrier can be modulated by changing material fabrication parameters [53].

It is worth noting that the clinical literature is divided on the wisdom of using BMP-2. While studies exist saying that it is safe and efficacious [59], other studies point out its limitations [60,61]. Based on current clinical best practices, the concentration of BMP-2 needed to achieve a useful response is much higher than the naturally occurring concentration of BMP-2. This raises concerns about carcinogenicity. In addition, there are important site-specific dosing concerns regarding ectopic bone formation and neurological complications that make it necessary to test a product in each location of its intended use. This can be costly and delay time to market for new products using this growth factor. Recognizing these complexities, we moved forward to test our material as a BMP-2 carrier.

**Figure 10:**
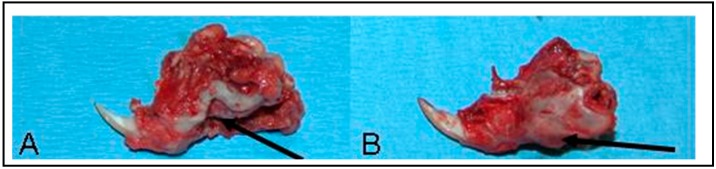
Explanted left hemimandibles (with soft tissue attached) in the experimental groups at the conclusion of an 8 week study. (**A**) Undoped nanophase composite implanted in the critical size defect (arrow) and (**B**) BMP-2 doped nanocomposite implanted in a critical size defect with robust bony ingrowth (arrow).

**Figure 11:**
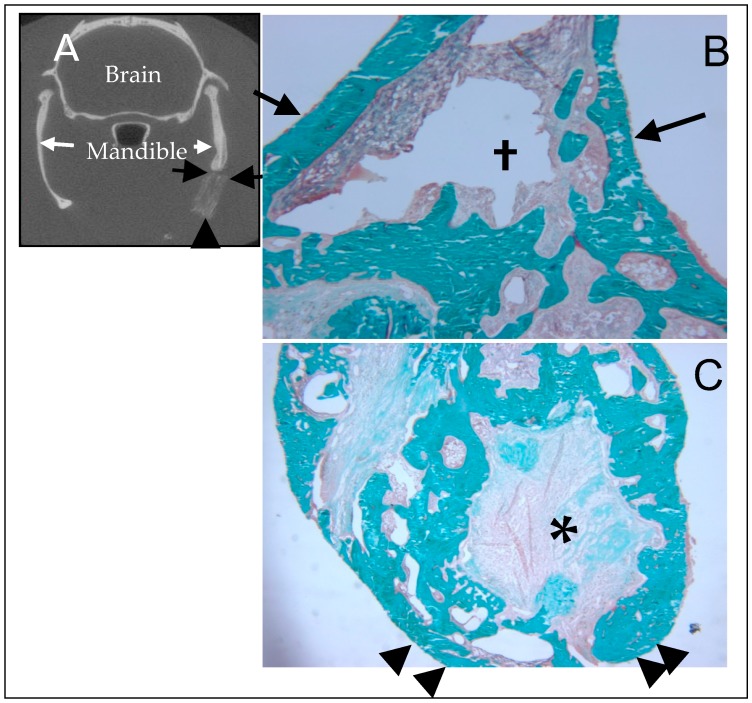
Histological evaluation of the BMP-2-doped NBS from Figure 10. The non-demineralized sections are embedded in acrylic and stained with Goldner’s trichrome. Mineralized tissue stains blue. (**A**) This coronal μCT section provides orientation for B & C. The black arrows point to the NBS/bone interface (corresponding to those in (**B**)) with the implant seen beneath the arrows. The arrow head indicates the inferior border of the NBS (corresponding to those in (**C**)). (**B**) The arrows demonstrate the location of the original interface between mandible and NBS. Note complete osteointegration of the NBS and robust bone formation. † The open space is due to a processing artifact (40×). (**C**) The defect volume consists primarily of new bone. There remains NBS material in the core (indicated with *), but the interface between bone and NBS is irregular and marked by active bone remodeling without fibrous tissue. The area marked with the arrow heads is the free inferior border of the mandible/implant (20×). This figure presented with permission from Wiley [38].

**Figure 12:**
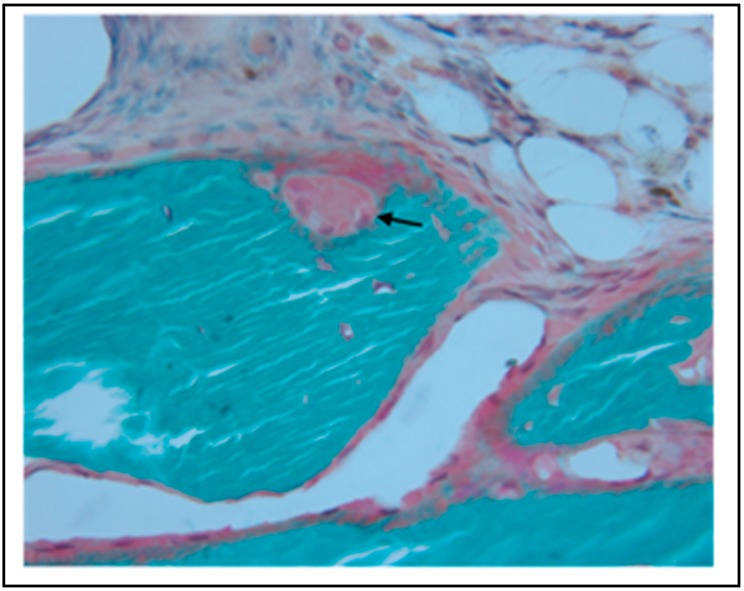
Osteoclastic activity (arrow) was noted throughout the bulk of the implant and indicates normal remodeling of the implant at 8 weeks.

In a study evaluating our material combined with BMP-2, non-crosslinked samples were tested by applying a bolus of 3 μg BMP-2 in 30 μL DI water directly to the implant 15 min prior to implantation (allowing absorption by capillary action). This dosage was based on prior studies in the rat mandible [62]. The method of dosing the materials with BMP-2 was intended to mimic the clinically used process associated with Medtronic’s Infuse^TM^ product. In an 8-week study, the samples exhibited good osteointegration with excellent spanning of the defect with hard callus (Figure 10). Mechanical strength and stiffness of the implanted hemimandibles closely approximated the contralateral unoperated side. Histologically, the material showed no significant evidence of a foreign body reaction. Complete osteointegration with no discernable border between implant and native bone was seen (Figure 11). In addition, there was clear evidence of active bone remodeling within the implant (Figure 12) [63]. Some organized fibrous tissue at the core of the implant was presumably an area not infiltrated by the BMP-2. In contrast, heavily crosslinked samples exhibited poor osteointegration. This is likely related to poor diffusion of the BMP into the denser material, as well as poor biological properties associated with crosslinking. These results demonstrate three important points. First, the subcutaneous model was predictive of the bony model with respect to material-host interface and material stability. Second, the material is an effective carrier for BMP-2 when the protein is properly absorbed into the material. Finally, the material needs some degree of crosslinking to confer necessary mechanical properties, yet high levels of crosslinking interfere with BMP-2 uptake and therefore osteointegration.

Based on the combined results from the previous paragraph and the section above entitled “BMP-2 Release”, we construct the following argument. Effective doping of BMP into scaffold materials can be enhanced in crosslinked samples by taking advantage of scaffold architecture. Aside from chemical interaction, another mechanism for preserving the protein in a carrier and releasing it in delayed fashion is physical hindrance [53,64,65]. Given the spatial dimensions of the BMP-2 molecule [66], a steric interdigitation of the molecule into the gap zones between collagen monomers of the collagen fibril appears to be feasible. This can be done during fibrillogenesis. This theory assumes that BMP release will be controlled by in vivo enzymatic scaffold degradation as part of the bone healing response. By combining the nanoscale 3D architecture of the collagen/mineral bone fibril with steric interdigitation of BMP-2, densification, and mild crosslinking, we have evidence to suggest that we can produce a new bone substitute material that resists moderate compressive, shear, and tensile forces while activating new bone formation. There is no comparable material currently available for clinical skeletal reconstruction.

## 8. Summary

The nanophase bone substitute (NBS) is a composed of a composite scaffold engineered at the nanoscale. It is osteointegrative and has the potential for robust mechanical stability in a physiological environment. Furthermore, the synthesis process can be tailored to encourage biologically induced bone morphogenetic protein release instead of less regulated physical diffusion. Thus, it can be used as a carrier of growth/differentiation factors and cytokines, when enhanced osteoinductive properties are useful. We view the NBS as a platform technology with broad ranging clinical potential. Its obvious application is in reconstructing traumatic bony defects, as well as those that exist because of congenital malformation. If used with cytokines or growth factors, it would not be suitable for post-cancer extirpative reconstruction due to the concern for tumorgenicity. However, we envision a possible use for the NBS in a post-neoplasm environment when used as a carrier for antineoplastic agents. The study of the NBS in the rat mandible presented here focused on the craniofacial skeleton. Ultimately, the goal is to produce a fully bio-degradable bone substitute material that can be used to reconstruct bony defects in any part of the skeleton that requires initial implant stability before being replaced by bone.

## Abbreviations

BMP-2	Bone morphogenetic protein 2
Ca/P	Calcium/phosphate
CSD	Critical size defect
BM	Demineralized bone matrix
DI	Deionized
EDX	Energy dispersive x-ray analysis
ELISA	Enzyme-linked immunosorbent assay
FBGC	Foreign body giant cell
HA	Hydroxyapatite
H&E	Hematoxylin and eosin
IACUC	Institutional animal care and use committee
NBS	Nanophase bone substitute
PLA/PGA	Polylactic acid/polyglycolic acid
SEM	Scanning electron microscopy
TCP	Tricalcium phosphate
TEM	Transmission electron microscopy
TRAP	Tartrate resistant acid phosphatase
UP	Uniaxially pressed
